# Fatty acid analyses provide novel insights on hippo defecation and consequences for aquatic food webs

**DOI:** 10.1038/s41598-020-68369-5

**Published:** 2020-07-21

**Authors:** Jessica Dawson, Deena Pillay, Renzo Perissinotto, Nicole B. Richoux

**Affiliations:** 1 Department of Biological Sciences, Marine Research Institute, University of Cape Town, Cape Town, 7701 South Africa; 2 Institute for Coastal and Marine Research (CMR), Nelson Mandela University, Port Elizabeth, 6031 South Africa; 3 Department of Zoology and Entomology, Rhodes University, Grahamstown, 6139 South Africa

**Keywords:** Ecosystem ecology, Ecology, Ecology

## Abstract

By defecating grasses into aquatic systems at massive scales and intensities, hippos can initiate complex changes to aquatic ecosystems. However, consequent effects on food webs are not well understood, particularly regarding shifts in basal resource contributions to consumer diets and their physiological condition. Here, we use fatty acid analysis to show that dense hippo aggregations and high dung loading are associated with (1) alterations to basal resource pools, (2) reduced quality of sediment organic matter and (3) increases in terrestrial and bacterial biomarker levels, but declines in those of diatoms in estuarine secondary consumers. While hippo defecation can increase boundary permeability between terrestrial and aquatic systems, our findings indicate that this may lead to a shift from a microphytobenthic food web base to one with increasing bacterial contributions to higher consumers. Our findings expand understanding of the mechanisms by which an iconic African megaherbivore indirectly structures aquatic ecosystems.

## Introduction

The functioning of aquatic ecosystems depends critically on connectivity with adjacent habitats, with the flow of trophic resources across habitat boundaries being particularly important^1^. Mobile consumers are highly effective in mediating connectivity and resource flows^2^, but none are capable of matching the degree to which hippos (*Hippopotamus amphibious*) achieve this on the African continent. Through nightly foraging, hippos engineer intricate trail networks that connect vegetated landscapes with aquatic systems, resulting in reciprocal transfers of both biotic and abiotic materials^3,4^. Similarly, hippo movements engineer elaborate channel systems that extend aquatic ecosystems into terrestrial habitats^4,5^. However, the direct transfer of basal trophic resources through defecation is arguably the most influential mechanism by which hippos mediate connectivity and material flows across terrestrial-aquatic corridors, given the frequency, intensity and spatial scales over which this process occurs. Hippo populations translocate roughly 36 tons of grass into the Mara River system daily^6^, while annual inputs of 5,840 tons have been estimated for Lake Naivasha (Kenya)^7^. Based on these estimates, it is doubtful whether other natural processes could replicate the scale of hippo-mediated resource transfers from terrestrial to aquatic ecosystems.

Hippo-mediated dung-loading can initiate complex and sometimes counter-intuitive changes to aquatic ecosystems, particularly at high defecation rates and during low flow conditions. Specifically, studies have shown that dung inputs can lead to degradation of water quality, declines in primary and secondary productivity, and mass mortality of consumers in extreme cases^6,8–12^. Collectively, these studies have challenged the notion that dung inputs act as subsidies in aquatic ecosystems by strengthening bottom-up trophic processes and facilitating increases in consumer biomass/abundance^13–15^. At the level of food webs, however, significant knowledge gaps prevail. Studies based on stable isotope data have shown that hippo dung is distinct from other resources and can be traced through to consumer tissue^7,13^. This has led to suggestions that hippo dung may subsidise aquatic consumers^13–15^. Beyond these ideas, little is understood about the ecological pathways by which hippo defecation can influence aquatic food webs and ecosystem functioning.

Poorly appreciated currently is that once voided, hippo dung can alter aquatic food webs via functionally distinct pathways that are independent of the role of dung as a trophic resource. In effect, once dung enters aquatic ecosystems, it can modify conditions that influence autochthonous production^8^. For example, when voided in copious amounts, dung can induce light limitation that leads to declining primary production^8,11^ and its contribution to food webs. Dung can concurrently function as a substrate for bacteria and other microbes^16^, which can be incorporated into food webs through consumption. It is therefore plausible that at high levels, defecation by hippos can induce shifts at the base of aquatic food webs, particularly by increasing dietary contributions of bacteria and decreasing those of autotrophs. There is also potential for dung inputs to reduce the physiological condition of consumers, since dung comprises mainly poor quality C_4_ grasses^17,18^. In addition, shading-induced reductions in autotrophic production and increases in that of bacteria may further contribute to decreased consumer health, given that bacteria are typically less nutritious than autotrophs^19–22^.

In this paper, we utilise fatty acid analyses to determine the effects of hippo dung inputs on food webs in the St Lucia Estuary, which is Africa’s largest estuarine lake^23^. The system supports roughly 1,000 hippos, which is estimated to be growing by 2–3% per year^24^. We utilise a comparative in situ approach to determine differences in fatty acid profiles and biomarker levels in aquatic consumers inhabiting biotopes with contrasting hippo densities and hence dung-loading. Fatty acids are a diverse group of carbon-rich compounds that occur in all organisms and are responsible for membrane structure and energy storage^25–27^. Due to their metabolic stability, even after being consumed^26,28^, fatty acids are highly effective biological tracers that can shed light on consumer-resource relationships^25^. In addition, specific fatty acid biomarkers can provide critical information on the relative importance of dietary resources for consumers, pathways by which basal resources are incorporated into food webs and the physiological condition of consumers in relation to diet. For example, diatom^29–31^ and bacterial^32–35^ biomarkers provide information on the relative importance of these basal resources for consumers, while terrestrial markers^30,31,35^ shed light on the dietary importance of land-derived resources. Similarly, essential fatty acid (EFA) levels provide information on the physiological health of consumers based on the premise that consumption of high quality trophic resources leads to greater EFA quantities in consumers^22,34,36,37^. EFAs are not synthesized by animals, but are produced predominantly by algae, and are essential for consumers^34^. EFAs are obtained by animals almost exclusively through consumption of aquatic primary producers, directly or indirectly^34,38^.

We hypothesised that food web components in biotopes with high hippo densities and dung loading would have distinct fatty acid profiles relative to those in which hippos are rare. We secondly hypothesised that basal resource and consumer fatty acid profiles would be more similar to hippo dung in high-density hippo biotopes than those in which hippos were scarce. The final hypothesis tested whether consumers exhibit relatively greater terrestrial and bacterial biomarker levels but lower diatom and EFA levels in biotopes with high hippo density. The last hypothesis was based on the rationale that defecation of land-derived grasses in biotopes where hippos were abundant would lead to greater terrestrial signatures in consumers, while in situ decomposition and addition of gut bacteria would increase consumer bacterial biomarker levels. Additionally, we expected reduced benthic diatom signatures in consumers in hippo-dense biotopes due to light limitation induced by dung. We also expected consumer condition (measured using EFAs) to be reduced in the presence of dense aggregates of hippos due to the prevalence of terrestrial grasses and bacteria as basal resources, which are of low quality relative to aquatic producers such as diatoms^39–41^. This is mainly because aquatic primary producers, such as diatoms, typically have greater EFA levels relative to terrestrially derived food sources^34,42^. Data were collected over multiple seasons to determine whether spatial trends in fatty acids and biomarkers between biotopes with contrasting hippo densities were temporally consistent. Broadly, our study was motivated by a need to understand the role of hippo dung in estuarine ecosystems, given that most work on the topic has been based on freshwater systems.

## Methods

### Study site

**Figure 1:**
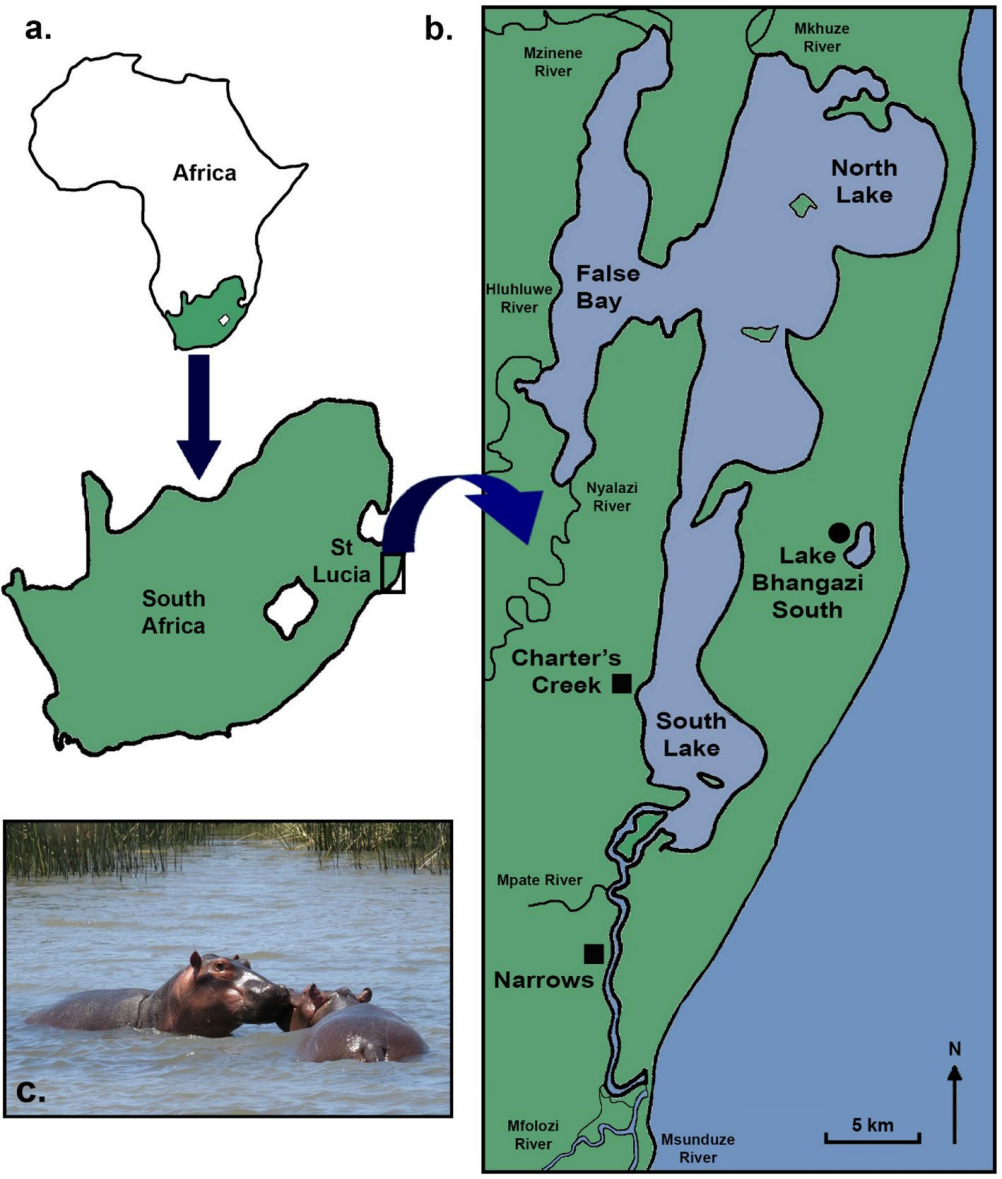
Map showing the geographical location of South Africa (**A**) and the St Lucia Estuary (**B**), along with sample collection sites (Narrows and Charter’s Creek). A pair of hippos in the Narrows, which contains roughly 50% of the St Lucia hippo population, is shown in (**C**). NB: (**B**) shows system boundaries and not water levels at the time of the study. Map produced using Adobe Photoshop CS6 (https://www.adobe.com/products/photoshop.html).

The St Lucia Estuary is a sub-tropical system located on the north-eastern coast of South Africa. It is a major component of the iSimangaliso Wetland Park, which is a UNESCO World Heritage Site^43^ (Fig. 1). The system typically undergoes cyclical droughts and flooding, with each phase lasting between 4 and 10 years^43^. At the time of sampling (2014–2015), the system was at the end of one of the most severe droughts recorded, resulting in major losses of aquatic habitat (up to 90% in the lakes) due to evaporation, water abstraction and decadal mouth closure from the Indian Ocean^23,43–46^. The estuary comprises three shallow (±1 m) interconnected lakes (South Lake, North Lake and False Bay), which together cover an area of roughly 328 km^2,47^. The lakes discharge into the Indian Ocean via a deeper (± 2 m) 22 km long, meandering channel called the Narrows (Fig. 1). Hippos are a dominant component of the ecosystem and have been estimated to annually translocate close to 2000 tons of terrestrial grasses into the system through defecation^24^.

### Sampling design

A comparative in situ approach was used to test the hypotheses underlying this study. This approach involved collection of temporally replicated food web sample types common to two biotopes (the Narrows and Charter’s Creek) that had contrasting hippo densities. Fifty percent of the St Lucia hippo population occurs in the Narrows (Fig. 1), while Charter’s Creek is less frequently utilised by hippos^24^. Recent aerial censusing indicated a hippo density of 20.6 individuals/km of shoreline in the Narrows, but only 1.4 individuals/km at Charter’s Creek^48^. Three sites, spanning a total distance of 2–3 km, were sampled in the Narrows. Each site was located in close proximity to a resident hippo pod (between 15 to 30 hippos per pod) and comprised three subsites, with two being ± 50 m upstream and downstream of the pod and one being adjacent to the pod (50 m away). Similarly, three subsites were sampled at three sites within Charter’s Creek, also spanning a total distance of 2–3 km (Fig. 1).

Food web components were collected from multiple trophic positions (sediment organic matter—SOM, particulate organic matter—POM, zooplankton, benthic macrofauna and dominant fish) at each subsite on four occasions (March, July, November 2014 and February 2015). However, due to receding water levels at the end of sampling and the absence of some taxa within some seasons, food-web components common to the Narrows and Charter’s Creek could not be consistently sampled during the study. Sediment cores (n = 3, pooled per subsite, depth = 1 cm, diameter = 2 cm) were collected for the determination of SOM. Microphytobenthos (MPB) was separated from SOM through the phototactic migration of diatoms^49–51^, which involved sediment cores being spread over trays, overlaying of mesh (500 µm) over the sediment surface and then covering by 3–5 mm sterilized sand (autoclaved at 450 °C). Sediment was exposed to a direct florescent light (6–8 h), while moistened with filtered (Whatman GF/F) estuary water. The mesh was lifted along with the upper layer of sand and migrated MPB, while the remaining sediment was used for SOM determination. Sediment samples were stored in foil envelopes and frozen at − 10 °C in the field and at − 80 °C upon returning to the laboratory. Where necessary, samples were acidified (2% HCl) and rinsed in distilled water to remove inorganic calcium carbonate (CaCO_3_). Sediment samples were lyophilized at − 60 °C for 24–48 h (VirTis Benchtop K). Roughly 1,500 mg of SOM samples were weighed (Mettler Toledo XP205 microbalance) and stored in lipid-cleaned 10 ml test tubes. MPB was separated from SOM samples to avoid confounding hippo dung effects on sediment organic matter with processes related to autotrophy.

A water sample (500 ml) was collected at each subsite for POM analysis and sequentially filtered (100 µm mesh, followed by pre-combusted GF/F filters). Filters were acidified with 2% HCl to remove inorganic CaCO_3_. Zooplankton samples were collected using an epibenthic D-sled (radius = 18 cm, mesh = 100 µm, n = 1 per subsite, trawl distance = 30 m), filtered (100 µm Nitex mesh), enclosed in foil envelopes and frozen. In the laboratory, POM and zooplankton filters were lyophilized for at least 24 h. Each POM sample was weighed and stored in lipid-cleaned test tubes (10 ml). Zooplankton samples were ground, and a 25 mg sub-sample used for analysis. Due to the potential for fatty acid tissue samples to degrade if not frozen, it was not feasible to sort the zooplankton samples into dominant taxa.

Benthic macrofauna samples collected with a Zabalocki-type Ekman grab (area = 0.026 m^2^; 2 pooled/subsite) were washed sequentially through 500 (five times) and 2000 µm (one time) sieves, and retained organisms frozen in foil envelopes within 12 h of collection. All individuals of taxa common to the Narrows and Charter’s Creek (*Grandidierella bonnieroides* [amphipod] and *Cyathura estuaria* [isopod]) were pooled per subsite, ground and weighed. Dominant fish species (*Oreochromis mossambicus* [tilapia], *Chelon dumerili* [mullet] and *Ambassis ambassis* [glassy]) were collected using a purse-seine net at each subsite within Charter’s Creek, while a cast-net (radius = 2 m) was used in the Narrows. For larger fish, muscle samples (2 × 2 cm, below dorsal fin) were frozen in foil envelopes, while smaller fish were frozen whole. Tissue samples were lyophilized for 24–48 h and homogenised to powder.

Hippo dung samples from five fresh (voided within 24 h) dung middens, were collected along hippo pathways adjacent to the Narrows in two seasons, under the guidance of a park ranger. Dung samples were not collected from Charter’s Creek due to the scarcity of hippos in this biotope. This was compounded by limited access to rangers that could protect researchers from crocodiles, buffalo and hippo (albeit in small numbers) that occur in Charter’s Creek.

### Fatty acid analysis

All samples were stored at − 80 °C, followed by homogenisation in a mortar and pestle or peeled (filtered materials). Lipids were extracted and fatty acid methyl esters (FAMEs) derived using a modified one-step method^52,53^. Specifically, our methods followed those of Richoux et al.^51^, which were based originally on Indarti et al.^50^. While Indarti et al. used a series of temperatures and extraction times and caproic acid as the internal standard, Richoux et al. used a single temperature (100 °C) and extraction time (30 min), while using nonadecanoic acid as the standard. Aliquots of tissue were added to test tubes (10 ml) containing 2 ml chloroform (CHCl_3_) and 0.01% butylated hydroxytoluene. An internal standard [10–20 µl of 6–8 mg of nonadecanoic acid (19:0) per 10 ml of CHCl_3;_ final concentration: 0.6–0.8 mg/ml] was added to each sample before test tubes were flushed with nitrogen, sealed with Teflon tape and stored at -20 °C. A mixture of sulphuric acid and anhydrous methanol (0.3:1.7 ratio) was added to samples before re-flushing with nitrogen, sealing with teflon caps, vortexing, sonicating in an ice bath (5 min) and heating (30 min, 100 °C). Once cooled to room temperature, samples were diluted with 1 ml ultrapure (milliQ) water and centrifuged (3 min; 3,000 rpm; Hettich EBA 20). The upper aqueous layer of the sample was discarded and the lower lipid layer containing FAMEs was dried (sodium sulphate), rinsed through a drying filter, concentrated under nitrogen gas and then suspended in hexane.

Gas chromatographic (GC) analysis of FAMEs was performed using an Agilent 7890 equipped with a ZB-Waxplus 320 column (30 m long × 0.32 mm internal diameter) and a flame ionization detector (FID) with helium as the carrier gas. Aliquots of sample were injected using a G7683 auto-injector into the GC oven under the following temperature programme: 70 °C for 1 min, raised to 170 °C at 40 °C/min for 4 min, and then to 250 °C for 4.5 min. Chemstation (version B.04.02; https://www.agilent.com/en/products/software-informatics/massspec-workstations/gc-msd-chemstation-software) was used to integrate and calibrate FAME peaks produced. Representative samples of each food web component were further analysed using a gas chromatography/mass spectrometer (GC/MS; Agilent Technologies 7000 GCMS-QQQ running Masshunter version 5.00 and the NIST 08 MS library; https://www.agilent.com/en/products/software-informatics/masshunter- suite/masshunter/masshunter-software-with-msd-chemstation-da/msd-chemstation-da) equipped with an identical column type and using the same temperature protocol as in the GC analyses. These samples, combined with comparisons of retention times produced by known external standards, were used to confirm identities of peaks produced by FID. By comparing FAME peak areas with those of the internal standard (19:0), FAME amounts per sample were quantified as a fatty acid concentration (mg/g dry mass). Quantitative values were then transformed into qualitative data and expressed as a percentage of the total fatty acids per sample.

Several fatty acid biomarkers were used to compare the food web components between the Narrows and Charter’s Creek. These biomarkers included (1) a terrestrial marker: sum 18:2ω6 & 18:3ω3^30,31,35^, (2) a bacterial marker: sum 15:0, 17:0 and all iso- and anteiso-branched chain fatty acids^32–35^, (3) an essential fatty acid (EFA) biomarker: sum 20:4ω6, 20:5ω3 & 22:6ω3^34, 37,42^ and (4) a diatom ratio marker: sum of all fatty acids containing 16 carbon atoms relative to the sum of all fatty acids containing 18 carbon atoms^29–31^. We were unable to use long-chain saturated fatty acids as terrestrial markers, as these were not prevalent in the dataset. However, fatty acid data derived from a variety of South African estuaries indicated that 18:2ω6 and 18:3ω3 are dominant in higher plants (but not in sediments, particulate matter or diatoms separated by phototaxis from sediments), and this marker has been useful in tracking the fate of higher plant material through aquatic food webs^54–56^. Terrestrial, bacterial and essential fatty acid biomarkers are expressed as a percentage of the total fatty acids. Diatom biomarker values are expressed as the ratio of Σ16 to Σ18 carbon fatty acids.

### Data analysis

All multivariate analyses were performed in PRIMER v6.1 (https://www.primer-e.com) with unstandardized and untransformed fatty acid percentage data. The hypothesis that fatty acid profiles of food web components would be different between the Narrows and Charter’s Creek was visually tested using non-metric multidimensional scaling ordinations (nMDS) and statistically assessed using PERMANOVA (permutational analysis of variance) based on Bray–Curtis similarity matrices. PERMANOVA analyses were based on a nested hierarchical design in which “biotope” (Narrows or Charter’s Creek) was nested within “season”. SIMPER (similarity percentages) was used to evaluate the hypothesis that food web fatty acid profiles would be more similar to hippo dung in the Narrows than Charter’s Creek. For this analysis, hippo dung samples from the two sampling seasons were pooled, given that they were statistically indistinguishable (PERMANOVA: pseudo F_1,9_ = 2.537, *p* = 0.260). SIMPER is typically used in community analysis to quantify differences in multivariate groupings using a Bray–Curtis similarly index^57^. In our study, similarity indices were generated using SIMPER to quantify similarity of hippo dung fatty acid profiles to food web components in the Narrows and Charter’s Creek. All univariate analyses were conducted using the data analysis platform R v3.3.3 (https://cran.r-project.org). Nested ANOVA (analysis of variance) was used to test the hypothesis that bacterial and terrestrial biomarker values in consumers would be greater in the Narrows relative to Charter’s Creek, but that diatom and essential fatty acid levels would be greater in the latter biotope. Data normality (Q-Q plots) and homogeneity of variances (Bartlett Tests) were assessed to meet the assumptions required for parametric testing. Where relevant, data were transformed prior to parametric testing (log x + 1 or square root, depending on response data).

### Ethics statement

Sampling was conducted in accordance with guidelines of the University of Cape Town. Approval of the research was granted by the University of Cape Town, Science Faculty Animal Ethics Committee (approval number 2013/v8/DP).

## Results

### Fatty acid profiles: Narrows versus Charter’s Creek

Fatty acid profiles of basal resources (POM & SOM) and consumers (zooplankton, macrofauna & fish) differed significantly between the Narrows and Charter’s Creek (nested PERMANOVA *p* = 0.001; Table 1). This pattern was generally consistent within sampling seasons (Supplementary Figs. 1–5). Changes in fatty acid profiles between seasons were evident only in POM (nested PERMANOVA pseudo F_3,65_ = 2.871; *p* = 0.010) and tilapia (*Oreochromis mossambicus*; nested PERMANOVA pseudo F_3,175_ = 2.816; *p* = 0.013; Table 1).

**Table 1:** Results of nested PERMANOVA analyses testing for differences in fatty acid profiles of food web components between seasons and biotopes (Narrows vs Charter’s Creek).

Type	Species	Season			Biotope		
		*F*	DF	*p*	*F*	DF	*p*
Basal resources	Particulate organic matter	2.871	3,65	**0.010**	9.677	4,65	**0.001**
	Sediment organic matter	1.326	3,61	0.279	27.007	4,61	**0.001**
Primary consumers	Zooplankton	1.500	3,64	0.125	30.511	4,64	**0.001**
	*Grandidierella bonnieroides* (A)	1.092	2,40	0.338	13.107	3,40	**0.001**
	*Cyathura estuaria* (I)	0.982	1,11	0.465	11.192	2,11	**0.001**
Secondary consumers	*Oreochromis mossambicus* (F)	2.816	3,175	**0.013**	14.879	4,175	**0.001**
	*Chelon dumerili* (F)	1.041	2,77	0.426	8.672	3,77	**0.001**
	*Ambassis ambassis* (F)	2.072	1,70	0.165	5.215	2,70	**0.001**

Significant values are shown in bold.

Letters in parentheses denote broad taxonomic groupings: *A* amphipod, *I* isopod, *F* fish.

*F* pseudo F-statistic, *p* = significance, *DF* degrees of freedom.

### Similarity of food web profiles to dung

SIMPER analyses indicated that in 73% of comparisons (19/26), similarities to dung were greater in the Narrows than in Charter’s Creek (Tables 2, 3). Similarity to dung in the Narrows was between 1.3 and 15.3% (mean = 5.4%) greater than that in Charter’s Creek. In the seven cases where Charter’s Creek samples were more similar to dung, similarity was between 0.2 and 6.4% (mean = 1.9%) greater than for the Narrows.

**Table 2:** Results of SIMPER analyses showing similarity in fatty acid profiles of basal resources from either the Narrows or Charter’s Creek to hippo dung for each sampling season.

Basal resource	Season	Biotope	Dissimilarity between biotopes	Similarity to dung	Difference
Sediment organic matter	1	Narrows	27.83	65.59	**8.95**
		Charter’s		56.64	
	2	Narrows	18.73	61.54	**10.89**
		Charter’s		50.65	
	3	Narrows	34.15	20.99	**2.08**
		Charter’s		18.91	
	4	Narrows	33.12	63.26	**15.30**
		Charter’s		47.96	
Particulate organic matter	1	Narrows	39.76	51.89	− 3.30
		Charter’s		55.19	
	2	Narrows	33.23	41.01	− 1.22
		Charter’s		42.23	
	3	Narrows	20.31	22.73	**2.18**
		Charter’s		20.55	
	4	Narrows	30.22	52.58	**9.09**
		Charter’s		43.49	

Difference = disparity in similarity between fatty acid profiles of dung and those of Narrows and Charter’s basal resources; positive bold values indicate greater similarity in the Narrows, negative values indicate greater similarity in Charter’s Creek. Season 1: March 2014, Season 2: July 2014, Season 3: November 2014, Season 4: February 2015.

**Table 3:** Results of SIMPER analyses showing similarity in fatty acid profiles of primary and secondary consumers from either the Narrows or Charter’s Creek to hippo dung for each sampling season.

Consumer group	Season	Biotope	Dissimilarity between biotopes	Similarity to dung	Difference
*Oreochromis mossambicus* (A)	1	Narrows	17.15	43.18	**4.93**
		Charter’s		38.25	
	2	Narrows	14.60	43.22	**2.69**
		Charter’s		40.53	
	3	Narrows	15.18	45.29	− 1.76
		Charter’s		47.05	
	4	Narrows	14.83	42.58	**1.35**
		Charter’s		41.23	
*Chelon dumerili* (A)	1	Narrows	22.78	40.76	**2.06**
		Charter’s		38.70	
	2	Narrows	22.12	40.10	**2.12**
		Charter’s		37.98	
	3	Narrows	17.71	41.55	− 0.39
		Charter’s		41.94	
*Ambassis ambassis* (A)	2	Narrows	14.41	41.82	− 0.17
		Charter’s		41.99	
	3	Narrows	14.45	43.93	**1.76**
		Charter’s		42.17	
*Grandidierella bonnieroides* (A)	1	Narrows	26.71	55.32	**4.22**
		Charter’s		51.10	
	2	Narrows	27.65	49.43	**2.86**
		Charter’s		46.57	
	4	Narrows	17.66	51.01	**1.32**
		Charter’s		49.69	
*Cyathura estuaria* (I)	1	Narrows	30.11	56.11	**9.22**
		Charter’s		46.89	
	4	Narrows	21.66	51.93	**2.10**
		Charter’s		49.83	
Zooplankton	1	Narrows	37.82	52.38	**12.47**
		Charter’s		39.91	
	2	Narrows	33.79	32.40	− 6.37
		Charter’s		38.77	
	3	Narrows	16.54	42.01	**7.26**
		Charter’s		34.75	
	4	Narrows	11.31	34.31	− 0.72
		Charter’s		35.03	

Difference = disparity in similarity between fatty acid profiles of dung and those of Narrows and Charter’s consumers; positive bold values indicate greater similarity in the Narrows, negative values indicate greater similarity in Charter’s Creek. Letters in parentheses denote broad taxonomic groupings: *A* amphipod, *I* isopod, *F* fish. Season 1: March 2014, Season 2: July 2014, Season 3: November 2014, Season 4: February 2015.

### Fatty acid biomarkers: Narrows versus Charter’s Creek

**Figure 2:**
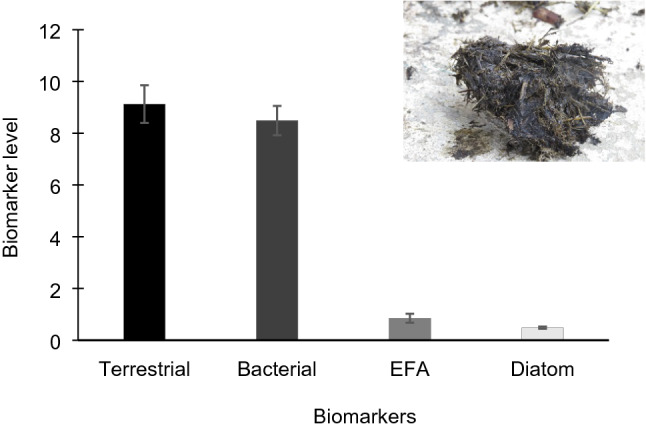
Mean biomarker values (± 1SE) for freshly voided hippo dung. Terrestrial, bacterial and essential fatty acid (EFA) biomarkers are expressed as a percentage of the total fatty acids. Diatom biomarker values are expressed as the ratio of Σ16 to Σ18 carbon fatty acids.

Freshly voided hippo dung had high terrestrial (9.13% ± 0.73 SE; Fig. 2) and bacterial biomarker values (8.49% ± 0.57 SE), but those for EFA and diatoms were substantially reduced (0.85 ± 0.18 SE and 0.48 ± 0.04 SE; Fig. 2). All four biomarker values for SOM and POM differed significantly between the Narrows and Charter’s Creek (nested ANOVA *p* < 0.001; Table 4) and with the exception of the diatom biomarker for SOM, showed significant seasonal variation (nested ANOVA *p* < 0.05). Different spatial trends for these basal resources were evident, with SOM bacterial values being greater in the Narrows in relation to Charter’s Creek, with the reverse trend being evident for diatoms (Supplementary Table 1). However, POM had greater terrestrial biomarker values in the Narrows than Charter’s Creek, but patterns of bacterial and diatom biomarkers were less clear, with hypothesised effects for these biomarkers being evident in two of the sampling seasons (seasons 2 & 3 for bacterial biomarkers, seasons 3 & 4 for diatom biomarkers; Supplementary Table 1). Patterns for terrestrial signatures in SOM were inconsistent, with higher values observed in the Narrows in the first two sampling seasons. Total EFA biomarker levels were consistently reduced in Narrows SOM samples relative to Charter’s Creek, with there being several cases in which this biomarker was not detected. This trend was not as apparent for POM, with EFA levels being reduced in the Narrows in the first two sampling seasons.

**Table 4:** Results of nested ANOVA testing the effects of season, biotope and site on basal resource and primary consumer fatty acid biomarkers.

	Season			Biotope			Site		
	*F*	DF	*p*	*F*	DF	*p*	*F*	DF	*p*
**Sediment organic matter**									
Terrestrial	52.56	3,18	** < 0.001**	12.84	4,18	** < 0.001**	2.789	14,18	**0.022**
Bacterial	3.65	3,18	**0.032**	58.81	4,18	** < 0.001**	1.476	14,18	0.216
EFA	7.05	3,18	**0.002**	80.09	4,18	** < 0.001**	4.867	14,18	**0.001**
Diatom	2.62	3,18	0.083	50.91	4,18	** < 0.001**	0.721	14,18	0.730
**Particulate organic matter**									
Terrestrial	4.18	3,22	**0.017**	10.25	4,22	** < 0.001**	3.172	14,22	**0.008**
Bacterial	127.84	3,22	** < 0.001**	126.46	4,22	** < 0.001**	1.527	14,22	0.182
EFA	42.12	3,22	** < 0.001**	18.43	4,22	** < 0.001**	8.414	14,22	** < 0.001**
Diatom	27.86	3,22	** < 0.001**	20.81	4,22	** < 0.001**	1.865	14,22	0.092
Terrestrial	1.30	1,1	0.458	18.25	2,1	0.163	0.585	4,1	0.739
***Cyathura estuaria (I)***									
Bacterial	0.16	1,1	0.760	25.01	2,1	0.140	6.554	4,1	0.284
EFA	10.02	1,1	0.195	3.13	2,1	0.371	1.113	4,1	0.603
Diatom	0.64	1,1	0.571	5.55	2,1	0.287	0.294	4,1	0.861
Terrestrial	3.98	2,10	0.054	69.22	3,10	** < 0.001**	7.237	10,10	**0.002**
***Grandidierella bonnieroides (A)***									
Bacterial	3.71	2,10	0.063	76.66	3,10	** < 0.001**	2.516	10,10	0.081
EFA	3.52	2,10	0.070	6.02	3,10	**0.013**	1.353	10,10	0.321
Diatom	26.37	2,10	** < 0.001**	17.49	3,10	** < 0.001**	1.181	10,10	0.399
Terrestrial	313.69	3,22	** < 0.001**	191.60	4,22	** < 0.001**	4.161	14,22	**0.002**
Bacterial	77.41	3,22	** < 0.001**	347.37	4,22	** < 0.001**	8.926	14,22	** < 0.001**
**Zooplankton**									
EFA	28.86	3,22	** < 0.001**	17.93	4,22	** < 0.001**	1.104	14,22	0.408
Diatom	99.92	3,22	** < 0.001**	61.20	4,22	** < 0.001**	4.442	14,22	**0.001**

Significant values are shown in bold.

Letters in parentheses denote broad taxonomic groupings: *A* amphipod, *I* isopod.

*EFA* essential fatty acids, *F* F-statistic, *p* significance, *DF* degrees of freedom.

While all biomarker values for zooplankton and the amphipod *Grandidierella bonnieroides* showed significant differences between biotopes (nested ANOVA *p* < 0.05; Table 4), trends in relation to the hypotheses posed were not always consistent (Supplementary Table 1). For zooplankton, terrestrial biomarker values were greater in the Narrows in the first and third sampling seasons, EFA levels were lower in the Narrows in the first two seasons and diatom values were greater in Charter’s Creek in seasons 1 and 3. Zooplankton bacterial signatures were two to three times greater in the Narrows than Charter’s Creek in the first three sampling seasons. For the amphipod *G. bonnieroides*, terrestrial biomarker values were generally greater in the Narrows than Charter’s Creek, with bacterial values being greater in the Narrows in two of the three sampling seasons (seasons 1 and 2), and diatom values greater in Charter’s Creek in two of three seasons (seasons 2 and 4). EFA values for *G. bonnieroides* were reduced in the Narrows in the first two sampling seasons.

**Figure 3:**
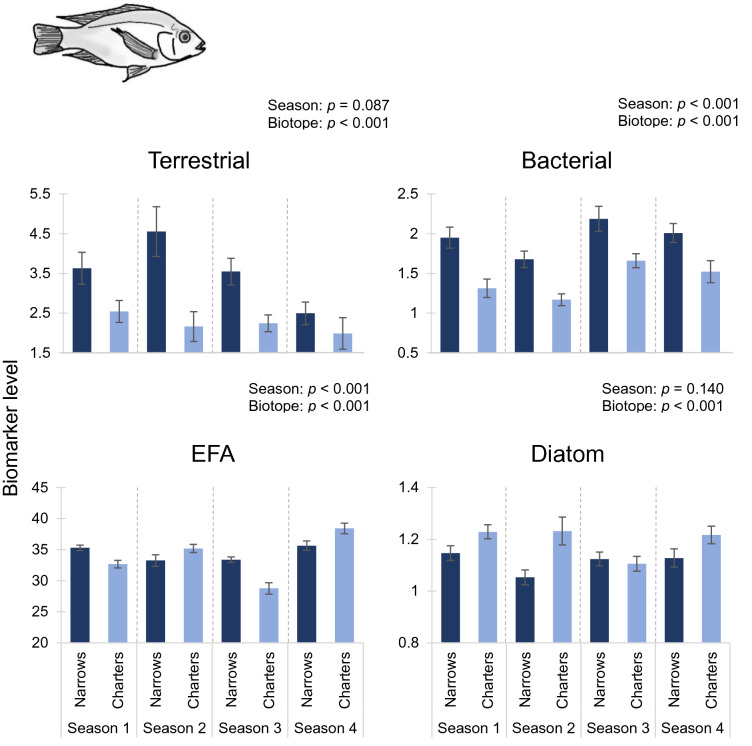
Differences in mean biomarker values (± 1SE) for tilapia (*Oreochromis mossambicus*) between the Narrows and Charter’s Creek over four sampling seasons. Terrestrial, bacterial and essential fatty acid (EFA) biomarkers are expressed as a percentage of the total fatty acids. Diatom biomarker values are expressed as the ratio of Σ16 to Σ18 carbon fatty acids. Season 1: March 2014, Season 2: July 2014, Season 3: November 2014, Season 4: February 2015. Results of statistical testing are presented in Table 5.

**Figure 4:**
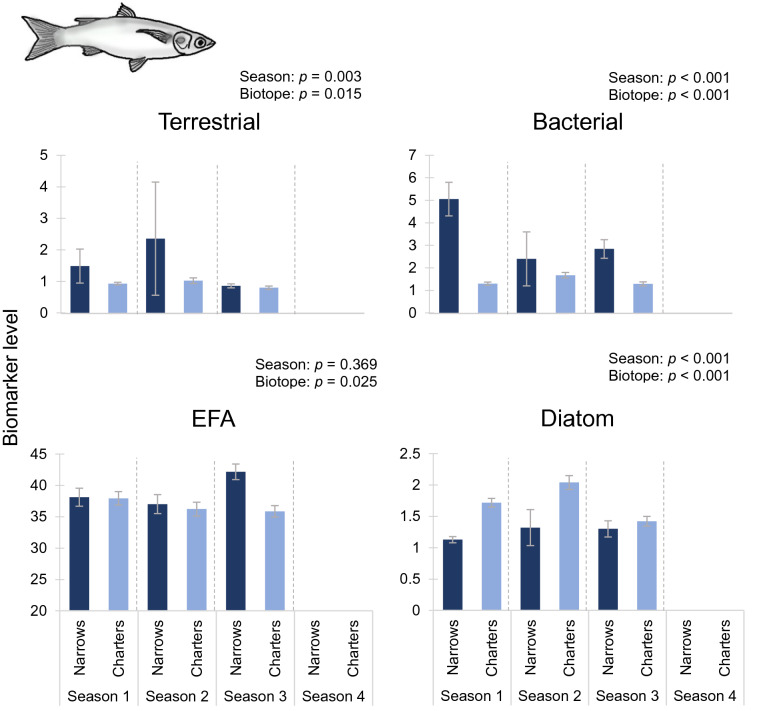
Differences in mean biomarker values (± 1SE) for mullet (*Chelon* (= *Liza*) *dumerili*) between the Narrows and Charter’s Creek over four sampling seasons. Terrestrial, bacterial and essential fatty acid (EFA) biomarkers are expressed as a percentage of the total fatty acids. Diatom biomarker values are expressed as the ratio of Σ16 to Σ18 carbon fatty acids. Season 1: March 2014, Season 2: July 2014, Season 3: November 2014, Season 4: February 2015. Results of statistical testing are presented in Table 5.

**Figure 5:**
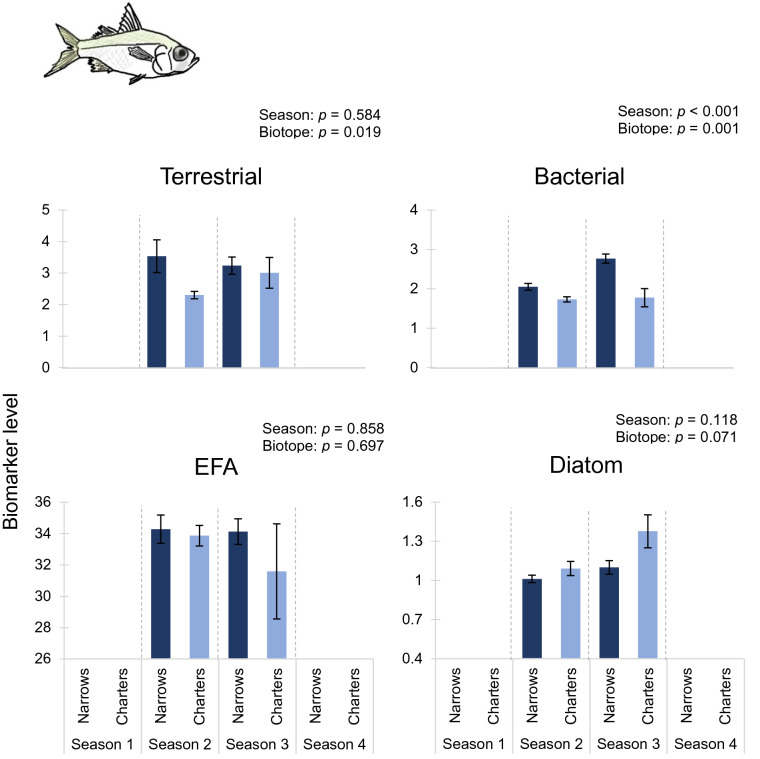
Differences in mean biomarker values (± 1SE) for glassy (*Ambassis ambassis*) between the Narrows and Charter’s Creek over four sampling seasons. Terrestrial, bacterial and essential fatty acid (EFA) biomarkers are expressed as a percentage of the total fatty acids. Diatom biomarker values are expressed as the ratio of Σ16 to Σ18 carbon fatty acids. Season 1: March 2014, Season 2: July 2014, Season 3: November 2014, Season 4: February 2015. Results of statistical testing are presented in Table 5.

Biomarker trends for higher consumers (fish) provided much stronger support for the hypotheses posed. With the exception of the EFA and diatom biomarkers for glassy (*Ambassis ambassis*), all markers for the fish differed between the Narrows and Charter’s Creek (nested ANOVA *p* < 0.001; Table 5). With minor exceptions, terrestrial, bacterial and diatom biomarker values followed hypothesised trends for tilapia (*Oreochromis mossambicus*; Fig. 3), with terrestrial and bacterial biomarkers being elevated in the Narrows, but diatom markers being greater in Charter’s Creek. Similar trends were recorded for mullet (*Chelon dumerili*; Fig. 4) and glassy (*Ambassis ambassis*; Fig. 5), though trends were less pronounced. Despite generally high terrestrial and bacterial signatures and low diatom values in the Narrows, there was little evidence of a decline in total EFA values in this biotope over the four sampling seasons (Figs. 3–5). Sample sizes of fish used in the fatty acid analysis are shown in Supplementary Table 2.

**Table 5:** Results of nested ANOVA testing the effects of season, biotope and site on secondary consumer (fish) fatty acid biomarkers.

Fish species	Biomarker	Season			Biotope			Site		
		*F*	DF	*p*	*F*	DF	*p*	*F*	DF	*p*
*Oreochromis mossambicus*	Terrestrial	2.23	3,133	0.087	9.54	4,133	** < 0.001**	6.259	14,133	** < 0.001**
	Bacterial	10.50	3,133	** < 0.001**	12.00	4,133	** < 0.001**	1.759	14,133	0.051
	EFA	21.47	3,133	** < 0.001**	13.31	4,133	** < 0.001**	5.427	14,133	** < 0.001**
	Diatom	1.86	3,133	0.140	5.53	4,133	** < 0.001**	1.296	14,133	0.218
*Chelon dumerili*	Terrestrial	6.75	2,49	**0.003**	3.82	3,49	**0.015**	7.183	11,49	** < 0.001**
	Bacterial	13.75	2,49	** < 0.001**	59.71	3,49	** < 0.001**	3.162	11,49	**0.003**
	EFA	1.02	2,49	0.369	3.41	3,49	**0.025**	1.29	11,49	0.258
	Diatom	16.62	2,49	** < 0.001**	8.84	3,49	** < 0.001**	1.259	11,49	0.276
*Ambassis ambassis*	Terrestrial	0.30	1,52	0.584	4.29	2,52	**0.019**	6.601	7,52	** < 0.001**
	Bacterial	46.12	1,52	** < 0.001**	7.85	2,52	**0.001**	0.939	7,52	0.485
	EFA	0.03	1,52	0.858	0.36	2,52	0.697	1.145	7,52	0.351
	Diatom	2.53	1,52	0.118	2.78	2,52	0.071	2.912	7,52	**0.012**

Significant values are shown in bold.

*EFA* essential fatty acids, *F* F-statistic, *p* significance, *DF* degrees of freedom.

## Discussion

Our findings from the St Lucia Estuary, which supports one of South Africa’s largest hippo populations that is growing by 2–3% per year^24^, provide novel insights on the potential for dung-loading by hippos to influence food webs and the functioning of estuarine and other similar aquatic ecosystems. We specifically aimed to test the following hypotheses: (1) food web components in biotopes with high hippo densities and dung loading have distinct fatty acid profiles relative to those in which hippos are rare; (2) basal resource and consumer fatty acid profiles are more similar to hippo dung in high-density hippo biotopes than those in which hippos were scarce and (3) consumers exhibit relatively greater terrestrial and bacterial biomarker levels but lower diatom and EFA levels in biotopes in which high hippos are abundant. Based on hippo densities and dung loading in the St Lucia Estuary, our findings suggest that persistent hippo defecation can influence the fatty acid composition of basal trophic resource pools (sediment organic matter [SOM], particulate organic matter [POM]) and higher consumers in particular, by increasing the incorporation of terrestrially-derived C_4_ grasses and bacterial resources into food webs, while reducing diatom contributions. Our data indicate that despite these changes in the Narrows, including lower essential fatty acid (EFA) levels of SOM, the condition of consumers did not decline significantly.

Fatty acid profiles of food web components were generally distinct between the Narrows and Charter’s Creek, sites which experience vastly different hippo occupation and therefore likely dung loading. Spatial differentiation in food webs has also been recorded using stable isotope data in freshwater studies, which showed distinct consumer isotopic compositions between sites with and without hippo dung^15^. Importantly, our findings from SIMPER analyses indicated that fatty acid profiles of food web components were generally more similar to dung within the Narrows than Charter’s Creek in 19 out of 26 comparisons. This result provides statistical support for the idea that differences in fatty acid composition between the Narrows and Charter’s Creek food webs were influenced by differences in dung loading.

Analyses of fatty acid biomarkers provided additional insights into the ecological pathways by which hippo-dung inputs can influence food web structure in aquatic ecosystems. It was expected that both SOM and POM from the Narrows would have greater terrestrial signatures relative to Charter’s Creek, given the low density of hippos in the latter biotope. Limited evidence supported this expectation for SOM, but for POM findings were strongly supportive. This contrasting outcome suggests that once voided, hippo dung is not necessarily incorporated into sediment matrices. This could be due to settled dung being rapidly decomposed into smaller, lighter particles that are either entrained and/or consumed in the water column. Bacterial biomarker values for POM were not consistently greater in the Narrows relative to Charter’s Creek, but this trend did emerge for SOM. This finding would suggest that once defecated, bacterial colonisation and breakdown of dung occurs predominantly in the sediment rather than in the water column, potentially because of greater physical stability in the benthos. Bacterial biomarker values that were recorded in the Narrows were almost double those reported in sediments from northern hemisphere lakes^30,35^, and were greater than those from intertidal sandflats in southern Japan^58^ and a detritus-rich inlet in the Sea of Japan^59^. Elevated bacterial signatures in the Narrows SOM were likely related to high dung loading, given the strong bacterial signature recorded in freshly voided hippo dung. For POM, spatial patterns in EFA biomarkers were inconsistent, but consistently large reductions in this marker in the Narrows suggest that hippo dung inputs may cause declines in the nutritional quality of SOM. This decline is likely driven by high bacterial contributions to SOM in the Narrows, but also reductions in diatom contributions. The latter finding aligns with our previous experimental work in the St Lucia Estuary, in which simulated enrichment of benthic plots with hippo dung (calibrated to volumes recorded in the Narrows) led to declines in microphytobenthic biomass by between 50 and 70% at the two experimental sites^8^.

Patterns in fatty acid biomarkers for higher consumers (the fish *Oreochromis mossambicus* and *Chelon dumerili*) strongly supported the hypotheses posed, with increased terrestrial and bacterial marker values, and decreased diatom values, in the Narrows relative to Charter’s Creek. For intermediate consumers (zooplankton and the amphipod *Grandidierella bonnieroides*), differences in biomarker levels between biotopes were less pronounced, and supported the hypotheses posed during some of the sampling seasons. It is difficult to explain the differences in biomarker patterns between intermediate and higher consumers, but these likely relate to variability in the feeding behaviour among consumers and their ability to assimilate and metabolise fatty acids. Despite feeding on prey that are dependent on terrestrially-derived resources, consumers may not express terrestrial fatty acids in their body tissues due to an inability to assimilate them^60^.

Differences in feeding traits and assimilation ability also likely explain variability observed in food web components within trophic positions. Terrestrial biomarker values for *C. dumerili* (mullet) were generally lower than values for *O. mossambicus* (tilapia) and *A. ambassis* (glassy). This finding suggests an inability by mullet to assimilate and express terrestrial biomarkers or that there are dietary differences among these fish. While both mullet and tilapia are iliophagous and consume detritus and small benthic animals^61,62^, tilapia are more reliant on detritus^61^. Therefore, the reduced terrestrial signatures in mullet suggest a lower contribution of dung, directly or indirectly, to their diets. Similarly, enhanced terrestrial signatures in the tilapia from the Narrows could indicate that this species, a known detritivore, is more reliant on dung.

It is recognised that EFAs can shed light on the condition or fitness of consumers based on the rationale that those that consume trophic resources of high quality would have greater quantities of EFAs^22,34,36,37^. Consumers gain more EFAs through the consumption of aquatic primary producers than terrestrial organic matter^22,42^, as there are high proportions of the EFAs 20:5ω3 and/or 22:6ω3 in diatoms and other algae^39–41^. As such, we expected that EFA levels in consumers would be reduced in the Narrows, due to high inputs of low-quality terrestrial grasses, relative to Charter’s Creek. In addition, lower diatom marker levels recorded in the Narrows, particularly in higher consumers, would have reinforced this expectation. However, consumer EFA levels were not consistently reduced in the Narrows, suggesting that levels of dung loading into the Narrows did not exceed a hypothetical threshold at which negative effects on consumer quality would manifest. Insignificant differences in consumer EFA levels between the Narrows and Charter’s Creek also suggest that alternative trophic resources were present in the Narrows, thus offering some resilience by allowing consumers to maintain nutritional quality despite dung loading by hippos. More broadly, our data may indicate that bacterial biomarkers (particularly for the fish) more effectively reflect the status of food web bases relative to EFAs.

While we did not detect consistent declines in the condition of consumers (according to total EFA levels) where hippo densities were high, dung loading by hippos at greater densities may conceivably lead to declines in consumer health. This may be the case in aquatic systems elsewhere in Africa where hippo population sizes are much greater than that of the St Lucia Estuary. For example, the Kenyan section of the Mara River has double the number of hippos relative to the St Lucia Estuary^10^. Flow reductions associated with droughts and water abstraction may cause additional declines in consumer quality in the long-term, by increasing dung retention and terrestrial and bacterial contributions. This may have been the case in the St Lucia Estuary during the 2008 drought, when roughly 90% of the lakes was lost due to evaporation. Under these conditions, it is plausible that copious dung inputs may have negatively influenced consumer quality through increased terrestrial and bacterial contributions. In addition, major diversity declines associated with the drought^43^ may have limited the availability of alternative trophic resources that could have countered declining consumer quality. Lastly, it must be borne in mind that the consumers sampled in the present study are likely to be highly resilient species^63,64^, given that our study was conducted at the tail-end of a severe dry phase that persisted for more than a decade.

Broadly, our findings provide evidence that dung inputs by hippos can influence fatty acid profiles of basal resource pools, while increasing terrestrial and bacterial biomarker contributions in higher consumers and decreasing those of diatoms. These findings are intuitive, given that dung comprises mainly terrestrial C_4_ grasses and has a strong bacterial signature. Observed reductions in diatom biomarker levels in higher consumers align with our previous experiments, which showed reductions in benthic microalgal biomass by up to 70% following in situ enrichment with hippo dung. The field and laboratory data together suggest a mechanistic link between hippo dung inputs and low diatom biomarker levels in fish in the Narrows. High levels of bacterial biomarkers have been linked with consumer ingestion of organic matter derived from coastal vegetation such as mangroves^58,65^. In the St Lucia Estuary, mangrove litter production during a period of artificial mouth opening (1980 to 1982) was estimated at 1,323 tonnes (dry weight) per year^24,66^. However, this is a very different picture to the drought- and abstraction-induced dry phase during which the current study was conducted. Near-decadal mouth closure from 2002 resulted in significant mangrove declines in the Narrows, followed by further declines in 2013/2014 caused by the reconnection of the Mfolozi River with the St Lucia Estuary^67^. Mangrove contributions to POM in the 1980s, when mangroves were more abundant, were just over half of the current estimated contribution of hippo dung of roughly 2000 tonnes of dry matter per year to the St Lucia Estuary^24^. Furthermore, local studies have documented small dietary contributions of mangrove material to estuarine fish relative to macro- and microalgae^68,69^. These lines of reasoning suggest that hippo dung is the major contributor to the detrital pool in the St Lucia Estuary, and the most likely explanation for greater levels of bacterial and terrestrial biomarkers recorded in the Narrows, particularly for the fish. Lastly, studies have demonstrated that under low-flow conditions, dung loading by hippos can lead to the development of hypoxia^9^, which can result in an enrichment of bacterial-derived fatty acids in consumer tissue relative to algal-derived fatty acids^70^.

Overall, our research has provided novel information on the potential food web ramifications of hippo dung inputs, while expanding perspectives on mechanisms by which these megaherbivores indirectly structure aquatic ecosystems, particularly in relation to altered basal resource contributions to consumers. Our work is relevant to ecosystems with large and growing hippo populations, such as protected areas, where dung loading at high intensities has the potential to increase bacterial and terrestrial contributions to basal resource pools, reduce autotrophic contributions and resource quality, and generate upward-cascading effects on higher consumers. We therefore suggest that further research on the relationship between hippo dung inputs and the nutritional quality of food web components, particularly under low-flow and high dung-loading conditions, would broaden understanding of the functional significance of hippo dung in aquatic ecosystems. Similarly, we suggest a need to further understand the implications of increased bacterial contributions for trophic interactions, food web dynamics and energy transfer through food webs to refine perspectives on the ramifications of hippo dung inputs for the functioning of aquatic ecosystems. This research path is relevant given suggestions that food webs with strong bacterial bases are less efficient and exhibit lowered consumer production^16,71^. At a broader level, our findings assist in shaping paradigms on the functioning of aquatic ecosystems prior to global megafaunal extirpations, including continent-wide declines of hippos. Recent research has suggested that periodic hypoxia associated with dung-loading by megaherbivores, including hippos, would have been a prominent feature of aquatic ecosystems prior to extinctions^10^. Similarly, our research suggests that terrestrial and bacterial contributions to aquatic food webs may have been greater than at present, given the major reduction in hippo abundance and distribution throughout much of Africa over the last century^72,73^ and that animal-mediated nutrient translocation has been estimated to have declined by 5–8% relative to the period prior to megafaunal extinctions in the late-Quaternary^74^.

## Supplementary information

Supplementary Figure Legends

Supplementary Tables and Figures
