# Supplementary material for: Fatty acid analyses provide novel insights on hippo defecation and consequences for aquatic food webs
Source: Sci Rep. 2020 Jul 21;10:12039. doi: 10.1038/s41598-020-68369-5 (PMC7374712; doi:10.1038/s41598-020-68369-5)
Supplement: Supplementary file 1 — Supplementary Figure Legends [file 41598_2020_68369_MOESM1_ESM.docx]

**Supplementary Figure Captions**

Supplementary Figure 1: Non-metric multidimensional scaling ordinations showing differences in basal resource fatty acid profiles between the Narrows and Charter’s Creek over the sampling period. SOM = sediment organic matter; POM = particulate organic matter. Season 1: March 2014, Season 2: July 2014, Season 3: November 2014, Season 4: February 2015.

Supplementary Figure 2: Non-metric multidimensional scaling ordinations showing differences in zooplankton fatty acid profiles between the Narrows (green) and Charter’s Creek (blue) over the sampling period. Season 1: March 2014, Season 2: July 2014, Season 3: November 2014, Season 4: February 2015.

Supplementary Figure 3: Non-metric multidimensional scaling ordinations showing differences in fatty acid profiles of the amphipod *Grandidierella bonnieroides* and the isopod *Cyathura estuaria* between the Narrows (green) and Charter’s Creek (blue) over the sampling period. Season 1: March 2014, Season 2: July 2014, Season 3: November 2014, Season 4: February 2015.

Supplementary Figure 4: Non-metric multidimensional scaling ordinations showing differences in fatty acid profiles of tilapia (*Oreochromis mossambicus*) between the Narrows (green) and Charter’s Creek (blue) over the sampling period. Season 1: March 2014, Season 2: July 2014, Season 3: November 2014, Season 4: February 2015.

Supplementary Figure 5: Non-metric multidimensional scaling ordinations showing differences in fatty acid profiles of mullet (*Chelon dumerili*) and glassy (*Ambassis ambassis*) between the Narrows (green) and Charter’s Creek (blue) over the sampling period. Season 1: March 2014, Season 2: July 2014, Season 3: November 2014, Season 4: February 2015.

Supplementary Figures


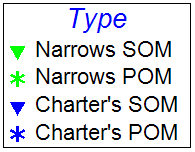


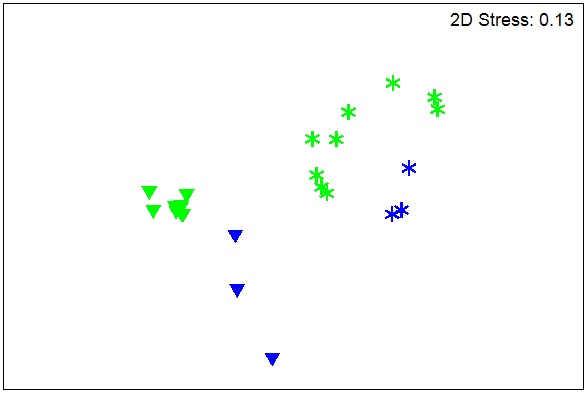

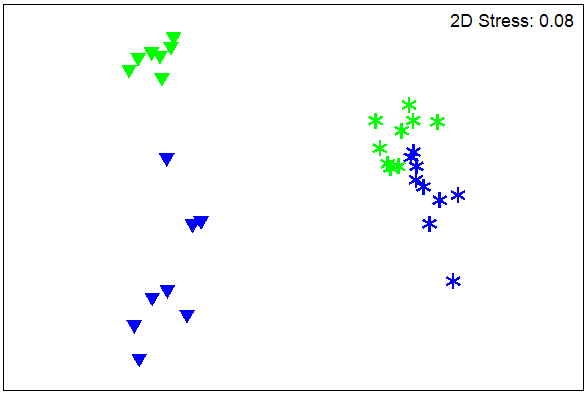

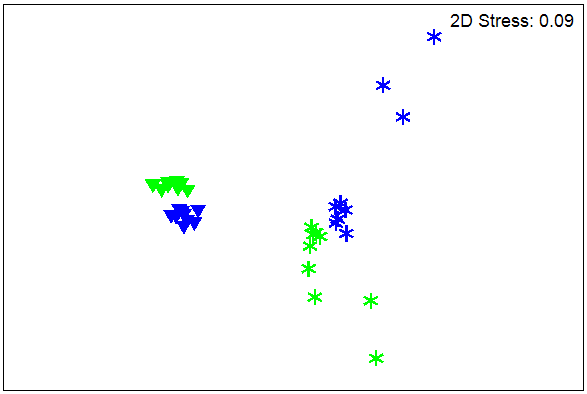

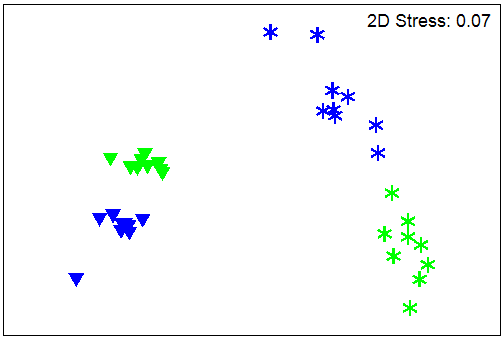


Season 4

Season 3

Season 2

Season 1

Supplementary Figure 1


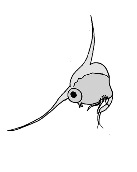

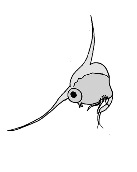

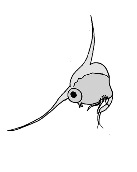

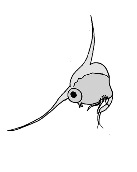

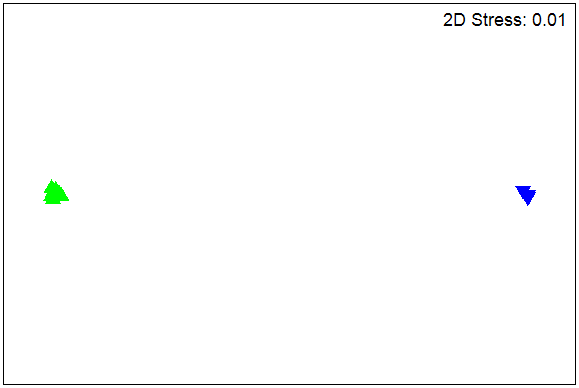

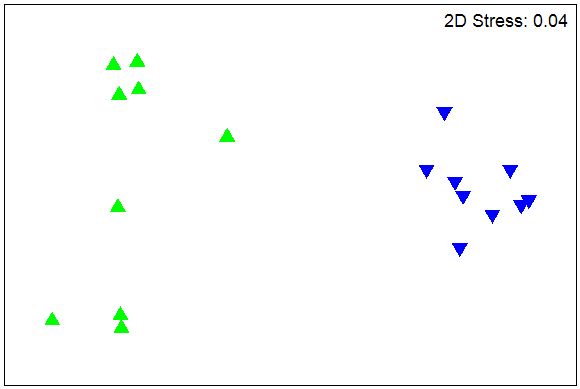

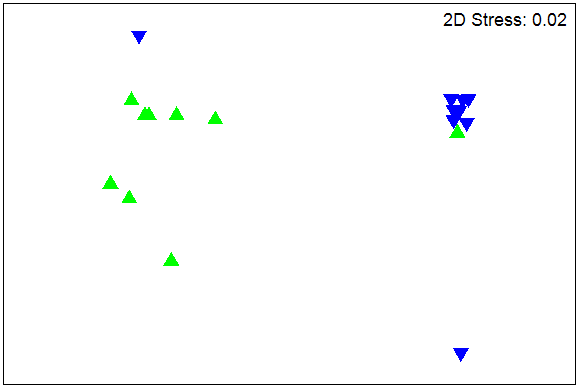

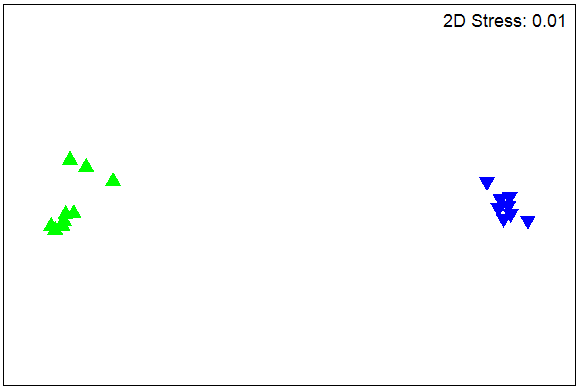


Season 4

Season 3

Season 2

Season 1

Supplementary Figure 2


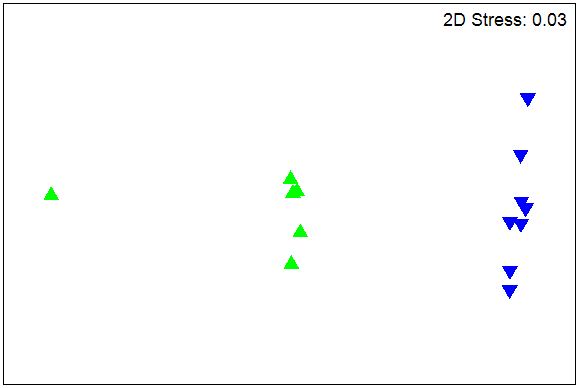

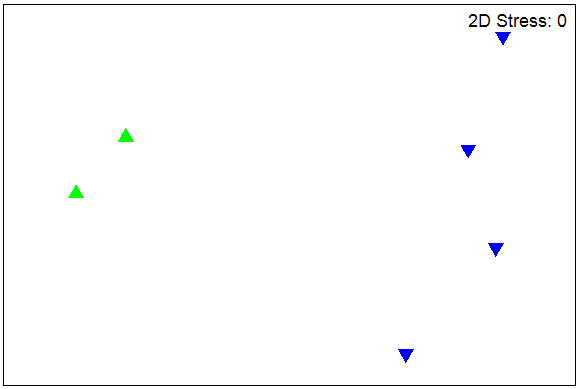

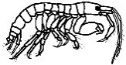

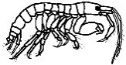

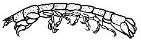


*Cyathura estuaria*

*Grandidierella bonnieroides*

Season 1

Season 1


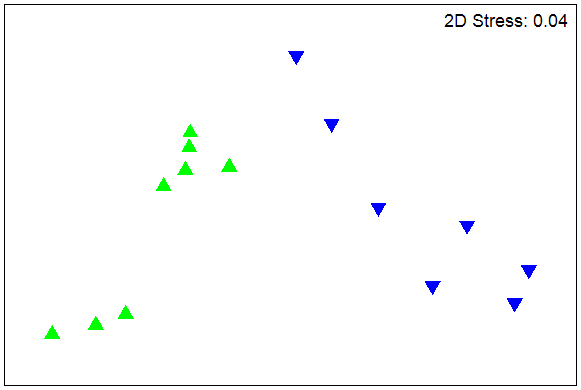


Season 2


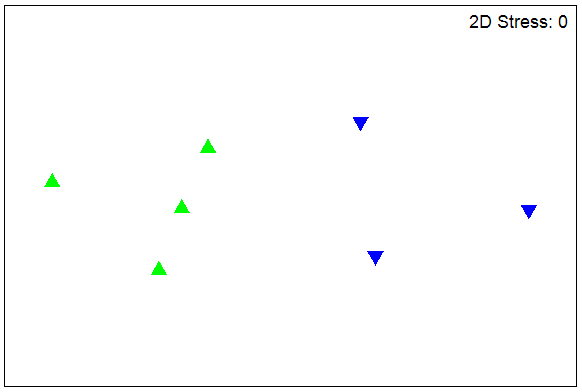

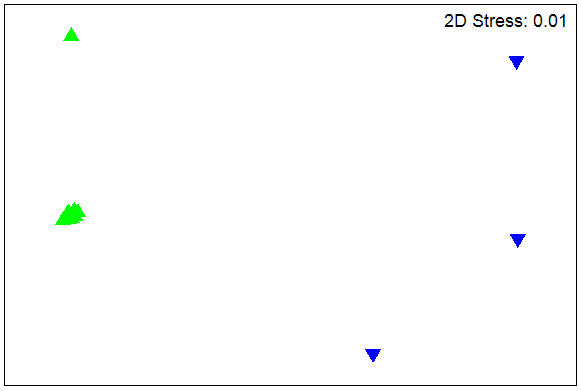


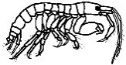


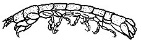


Season 4

Season 4

Supplementary Figure 3


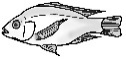


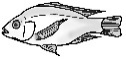


Season 2

Season 1

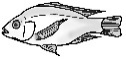

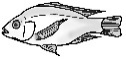


Season 3

Season 4

Supplementary Figure 4

*Chelon dumerili*


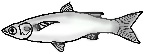

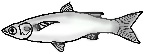

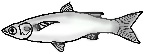


Season 1

*Ambassis ambassis*


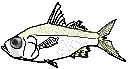

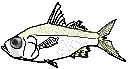


Season 2

Season 2

Season 3

Season 3

Supplementary Figure 5
