# Supplementary material for: Fatty acid analyses provide novel insights on hippo defecation and consequences for aquatic food webs
Source: Sci Rep. 2020 Jul 21;10:12039. doi: 10.1038/s41598-020-68369-5 (PMC7374712; doi:10.1038/s41598-020-68369-5)
Supplement: Supplementary file 2 — Supplementary Tables and Figures [file 41598_2020_68369_MOESM2_ESM.docx]

Supplementary Table Captions

Supplementary Table 1: Variation in mean biomarker values (± 1SE) for basal resource and primary consumers between the Narrows and Charter’s Creek over four sampling seasons. Terrestrial, bacterial and essential fatty acid biomarkers are expressed as a percentage of the total fatty acids. Diatom biomarker values are expressed as the ratio of Σ16 carbon FAs to Σ18 carbon fatty acids. Season 1: March 2014, Season 2: July 2014, Season 3: November 2014, Season 4: February 2015. Letters in parentheses denote broad taxonomic groupings: A = amphipod, I = isopod. ND denotes cases in which biomarkers were not detected.

Supplementary Table 2: Sample sizes of tilapia (*Oreochromis mossambicus*), mullet (*Chelon dumerili*) and glassy (*Ambassis ambassis*) used in fatty acid analysis. N = Narrows; CC = Charter’s Creek.

Supplementary Tables

Supplementary Table 1

| Resource | Biomarker |  | | | | | | | | | | | |
| --- | --- | --- | --- | --- | --- | --- | --- | --- | --- | --- | --- | --- | --- |
|  |  | Season 1 | | | Season 2 | | | Season 3 | | | Season 4 | | |
|  |  | Narrows | Charter's |  | Narrows | Charter's |  | Narrows | Charter's |  | Narrows | Charter's |  |
| Sediment organic matter | Terrestrial | 2.15 ± 0.26 | 1.21 ± 0.10 |  | 1.44 ± 0.06 | 1.22 ± 0.06 |  | 2.29 ± 0.48 | 3.65 ± 0.32 |  | 0.98 ± 0.09 | 0.97 ± 0.14 |  |
|  | Bacterial | 17.36 ± 0.57 | 14.83 ± 0.75 |  | 18.28 ± 0.58 | 14.8 ± 0.55 |  | 20.37 ± 0.35 | 10.79 ± 0.63 |  | 19.75 ± 0.70 | 10.05 ± 2.28 |  |
|  | Sum EFA | ND | 4.63 ± 0.47 |  | 0.53 ± 0.11 | 5.16 ± 0.95 |  | ND | 5.42 ± 0.70 |  | ND | 5.24 ± 2.10 |  |
|  | Diatom | 1.54 ± 0.05 | 2.87 ± 0.20 |  | 1.71 ± 0.06 | 2.89 ± 0.12 |  | 1.99 ± 0.08 | 2.5 ± 0.09 |  | 2.05 ± 0.04 | 4.07 ± 0.61 |  |
| Particulate organic matter | Terrestrial | 5.63 ± 0.93 | 3.53 ± 0.43 |  | 4.75 ± 1.03 | 3.76 ± 0.71 |  | 5.68 ± 0.50 | 2.88 ± 0.52 |  | 7.35 ± 0.59 | 2.67 ± 0.20 |  |
|  | Bacterial | ND | 10.65 ± 1.31 |  | 7.00 ± 0.40 | 1.68 ± 0.27 |  | 4.16 ± 0.39 | 5.51 ± 0.29 |  | 16.32 ± 1.21 | 13.09 ± 0.77 |  |
|  | Sum EFA | 4.79 ± 0.80 | 7.85 ± 0.94 |  | 5.78 ± 1.67 | 15.89 ± 3.89 |  | 13.06 ± 1.15 | 8.61 ± 0.69 |  | 2.16 ± 0.25 | 1.99 ± 0.65 |  |
|  | Diatom | 1.12 ± 0.12 | 1.38 ± 0.17 |  | 3.12 ± 0.35 | 1.72 ± 0.15 |  | 1.64 ± 0.09 | 3.23 ± 0.37 |  | 1.34 ± 0.09 | 2.65 ± 0.39 |  |
| Zooplankton | Terrestrial | 14.24 ± 0.45 | 3.93 ± 0.24 |  | 3.83 ± 0.60 | 6.58 ± 0.52 |  | 4.03 ± 0.13 | 2.54 ± 0.05 |  | 2.11 ± 0.04 | 2.44 ± 0.13 |  |
|  | Bacterial | 5.74 ± 0.14 | 2.46 ± 0.14 |  | 5.83 ± 0.58 | 1.99 ± 0.05 |  | 5.91 ± 0.26 | 2.19 ± 0.05 |  | 2.29 ± 0.06 | 2.38 ± 0.03 |  |
|  | Sum EFA | 23.75 ± 0.63 | 34.00 ± 0.54 |  | 18.04 ± 2.75 | 31.45 ± 3.57 |  | 36.01 ± 1.43 | 38.58 ± 0.46 |  | 34.09 ± 0.40 | 30.86 ± 0.81 |  |
|  | Diatom | 0.76 ± 0.01 | 1.78 ± 0.10 |  | 4.76 ± 0.57 | 1.85 ± 0.58 |  | 1.6 ± 0.04 | 2.55 ± 0.11 |  | 3.70 ± 0.05 | 3.41 ± 0.09 |  |
|  | Terrestrial | 7.34 ± 0.67 | 2.84 ± 0.08 |  | 5.30 ± 0.28 | 3.73 ± 0.61 |  |  |  |  | 4.02 ± 0.15 | 3.30 ± 0.20 |  |
| *Grandidierella bonnieroides* (A) | Bacterial | 10.58 ± 0.36 | 3.31 ± 0.27 |  | 11.92 ± 1.68 | 3.45 ± 0.45 |  | No Data | No Data |  | 6.99 ± 0.45 | 6.65 ± 0.87 |  |
|  | Sum EFA | 18.66 ± 2.43 | 27.37 ± 1.29 |  | 20.23 ± 2.85 | 25.55 ± 1.10 |  |  |  |  | 28.20 ± 0.56 | 24.70 ± 1.72 |  |
|  | Diatom | 0.69 ± 0.05 | 0.68 ± 0.03 |  | 0.87 ± 0.03 | 1.12 ± 0.06 |  |  |  |  | 0.65 ± 0.01 | 1.15 ± 0.13 |  |
|  | Terrestrial | 5.99 ± 0.22 | 1.97 ± 0.29 |  |  |  |  |  |  |  | 3.45 ± 0.23 | 1.89 ± 0.35 |  |
| *Cyathura estuaria* (I) | Bacterial | 6.35 ± 0.41 | 3.24 ± 0.79 |  | No Data | No Data |  | No Data | No Data |  | 3.91 ± 0.26 | 4.51 ± 0.30 |  |
|  | Sum EFA | 25.16 ± 3.80 | 22.96 ± 1.69 |  |  |  |  |  |  |  | 30.46 ± 1.12 | 25.71 ± 1.44 |  |
|  | Diatom | 0.49 ± 0.06 | 0.68 ± 0.06 |  |  |  |  |  |  |  | 0.54 ± 0.04 | 0.92 ± 0.10 |  |

Supplementary Table 2

|  | Season 1 |  | Season 2 |  | Season 3 |  | Season 4 |  |
| --- | --- | --- | --- | --- | --- | --- | --- | --- |
|  | N | CC | N | CC | N | CC | N | CC |
| Tilapia | 23 | 21 | 27 | 24 | 27 | 21 | 24 | 9 |
| Mullet | 8 | 13 | 3 | 27 | 6 | 21 |  |  |
| Glassy |  |  | 27 | 19 | 23 | 2 |  |  |


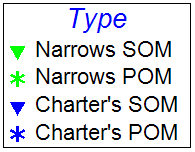
Supplementary Figures


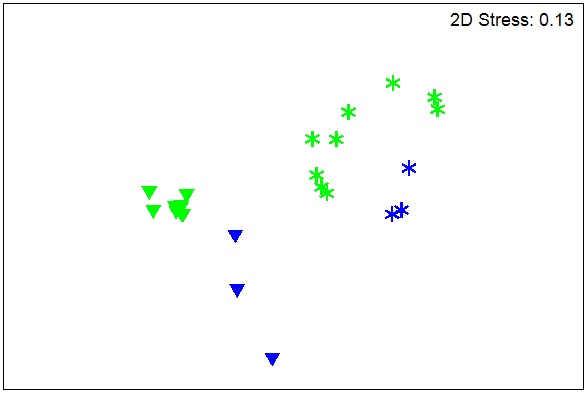

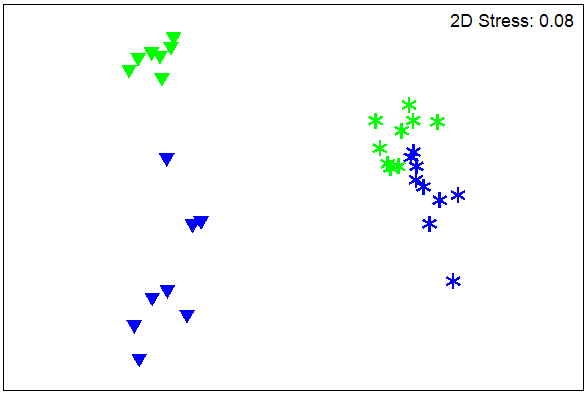

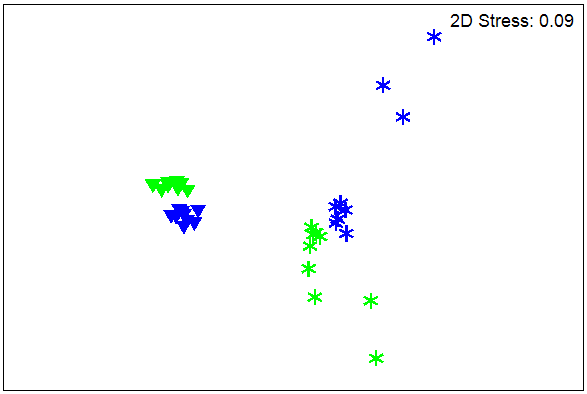

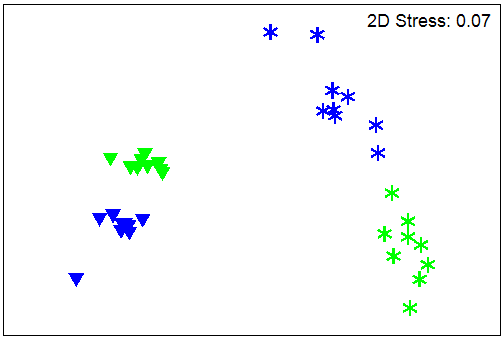


Season 4

Season 3

Season 2

Season 1

Supplementary Figure 1


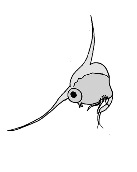

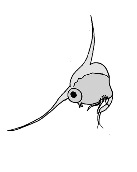

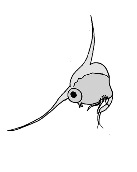

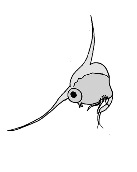

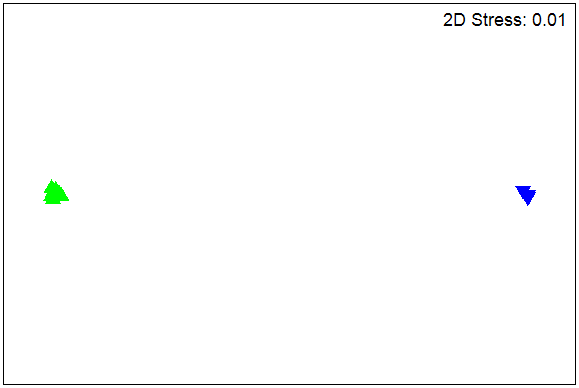

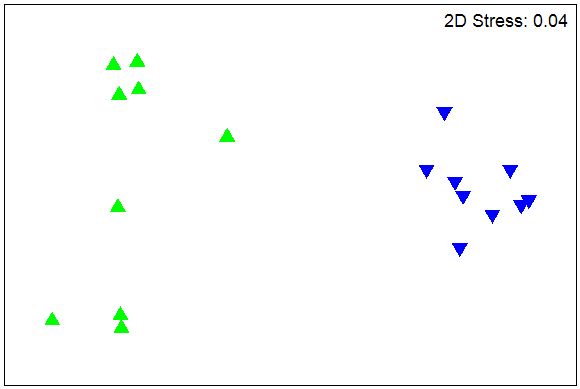

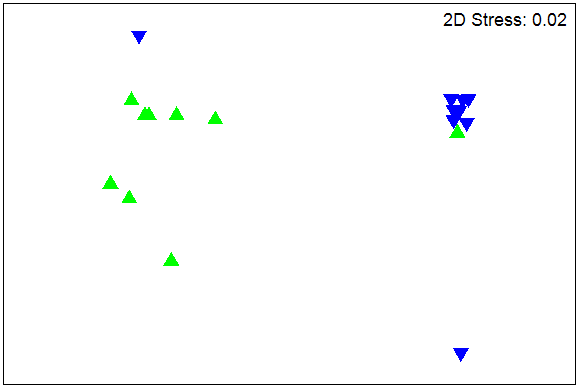

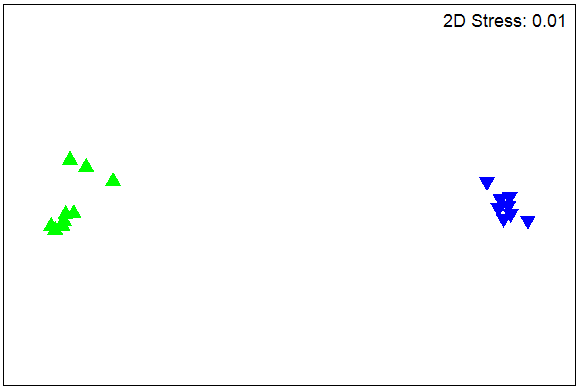


Season 4

Season 3

Season 2

Season 1

Supplementary Figure 2


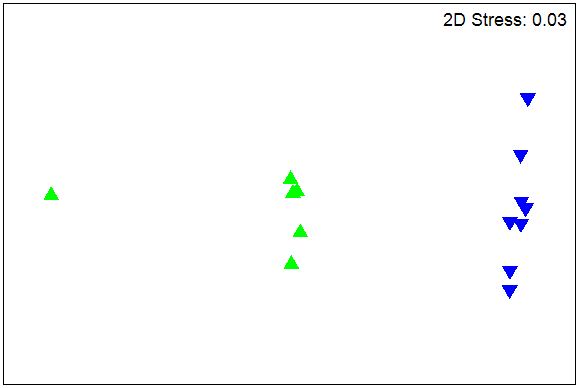

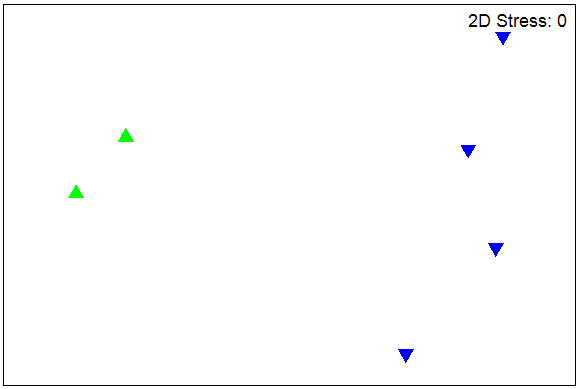

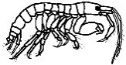

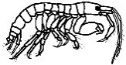

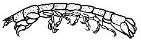


*Cyathura estuaria*

*Grandidierella bonnieroides*

Season 1

Season 1


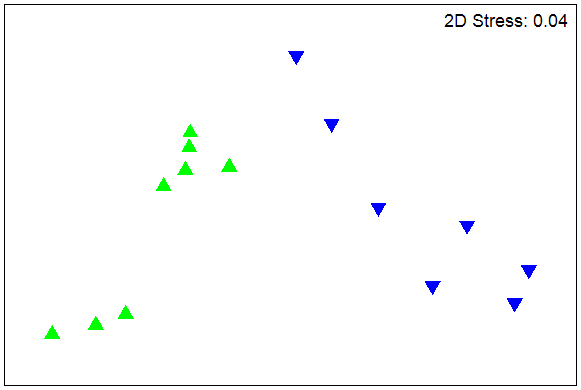


Season 2


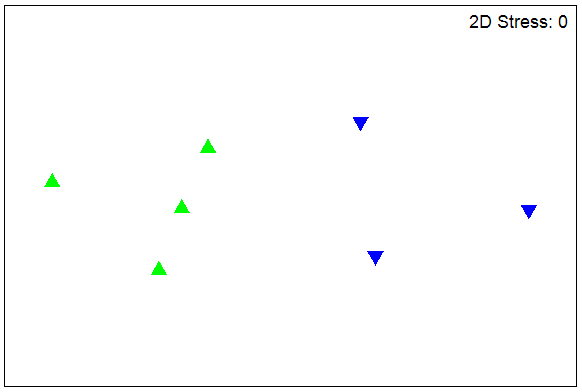

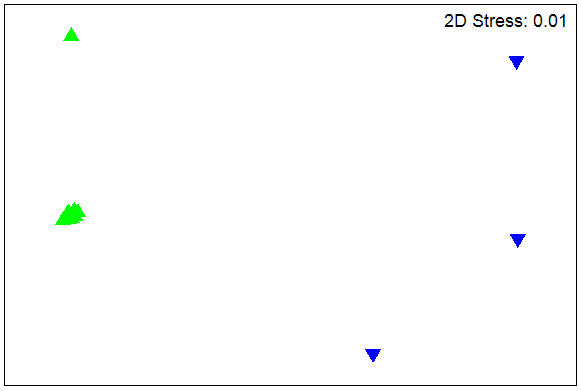


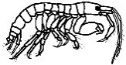


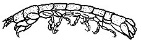


Season 4

Season 4

Supplementary Figure 3


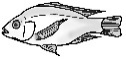


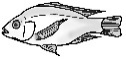


Season 2

Season 1

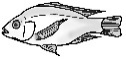

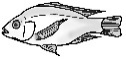


Season 3

Season 4

Supplementary Figure 4

*Chelon dumerili*


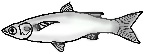

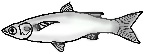

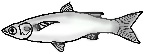


Season 1

*Ambassis ambassis*


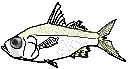

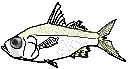


Season 2

Season 2

Season 3

Season 3

Supplementary Figure 5
